# Supporting Patient-Clinician Interaction in Chronic HIV Care: Design and Development of a Patient-Reported Outcomes Software Application

**DOI:** 10.2196/27861

**Published:** 2021-07-30

**Authors:** Susan Herrmann, Brad Power, Amineh Rashidi, Mark Cypher, Frank Mastaglia, Amy Grace, Elizabeth McKinnon, Pierre Sarrot, Christophe Michau, Matthew Skinner, Renae Desai, Martin Duracinsky

**Affiliations:** 1 Medical School University of Western Australia Crawley Australia; 2 College of Arts, Business, Law & Social Sciences Murdoch University Murdoch, WA Australia; 3 School of Nursing & Midwifery Edith Cowan University Joondalup, WA Australia; 4 School of Psychology and Exercise Science Murdoch University Murdoch, WA Australia; 5 Telethon Kids Institute Nedlands, WA Australia; 6 Patient-Centered Outcomes Research, Health Economics Clinical Trial Unit Hospital Hotel-Dieu, AP-HP University de Paris Paris France; 7 CeGIDD Centre Hospitalier Saint Nazaire Saint Nazaire France; 8 Sir Charles Gairdner Hospital Nedlands, WA Australia; 9 Perron Institute for Neurological & Translational Science Queen Elizabeth II Medical Centre Nedlands, WA Australia; 10 Department of Internal Medicine & Immunology Hospital Bicetre, AP-HP Kremlin-Bicetre France

**Keywords:** physician-patient communication, eHealth, primary care, ambulatory care, information systems, user-centered design, user context, patient reported outcomes, qualitative research, health care, HIV care, mobile phone

## Abstract

**Background:**

The consideration of health-related quality of life (HRQL) is a hallmark of best practice in HIV care. Information technology offers an opportunity to more closely engage patients with chronic HIV infection in their long-term management and support a focus on HRQL. However, the implementation of patient-reported outcome (PRO) measures, such as HRQL in routine care, is challenged by the need to synthesize data generated by questionnaires, the complexity of collecting data between patient visits, and the integration of results into clinical decision-making processes.

**Objective:**

Our aim is to design and pilot-test a multimedia software platform to overcome these challenges and provide a vehicle to increase focus on HRQL issues in HIV management.

**Methods:**

A multidisciplinary team in France and Australia conducted the study with 120 patients and 16 doctors contributing to the design and development of the software. We used agile development principles, user-centered design, and qualitative research methods to develop and pilot the software platform. We developed a prototype application to determine the acceptability of the software and piloted the final version with 41 Australian and 19 French residents using 2 validated electronic questionnaires, the Depression, Anxiety and Stress Scale-21 Items, and the Patient Reported Outcomes Quality of Life-HIV.

**Results:**

Testing of the prototype demonstrated that patients wanted an application that was intuitive and without excessive instruction, so it felt effortless to use, as well as secure and discreet. Clinicians wanted the PRO data synthesized, presented clearly and succinctly, and clinically actionable. Safety concerns for patients and clinicians included confidentiality, and the potential for breakdown in communication if insufficient user training was not provided. The final product, piloted with patients from both countries, showed that most respondents found the application easy to use and comprehend. The usability testing survey administered found that older Australians had reduced scores for understanding the visual interface (*P*=.004) and finding the *buttons* organized (*P*=.02). Three-fourths of the respondents were concerned with confidentiality (*P*=.007), and this result was more prevalent in participants with higher anxiety and stress scores (*P*=.01), as measured by the Depression, Anxiety and Stress Scale-21 Items. These statistical associations were not observed in 15 French patients who completed the same questionnaire.

**Conclusions:**

Digital applications in health care should be safe and fit for purpose. Our software was acceptable to patients and shows potential to overcome some barriers to the implementation of PROs in routine care. The design of the clinicians’ interface presents a solution to the problem of voluminous data, both synthesizing and providing a snapshot of longitudinal data. The next stage is to conduct a randomized controlled trial to determine whether patients experience increased satisfaction with care and whether doctors perceive that they deliver better clinical care without compromising efficiency.

## Introduction

### Background

Chronic HIV infection is a complex disease associated with psychosocial morbidity that can affect health-related quality of life (HRQL) detrimentally [1]; however, as clinical management in primary care settings often focuses on physical well-being and the outcomes of antiretroviral therapy [1], psychosocial and other relevant patient-volunteered information may not be engaged in the same clinically systematic manner. Challenges to implementing patient-reported outcome (PRO) measurements in routine care include the synthesis of voluminous data generated by questionnaires regardless of whether they are e-questionnaires or paper based, the complexity of collecting data between patient visits, and the integration of the results into clinical decision-making protocols [2]. There are additional organizational barriers to integrating PRO measurement in real-life settings, in hospitals and at home, but guidance is available [3]; and obstacles have also been recognized in localized settings [4]. As some barriers become surmountable [2], there is an increased impetus in fields, such as HIV and oncology, to use validated measures for the assessment of physical and psychosocial symptoms and quality of life beyond the hospital setting and between patient visits [5-7]. Muessig et al [7] identified opportunities for digital health strategies to be integrated within the HIV Continuum of Care to aid retention. Those concerned with cancer care and trials have taken advantage of the advances in digital communications [8] to configure hospital computer systems and capture PRO data in real time to better reflect the patient experience [9] and facilitate intervention. Kjaer et al [10] described the hospital-based implementation of a web-based tool to facilitate the use of PROs in HIV care. They adapted AmbuFlex [11], a generic web-based program for the collection and synthesis of PRO data, envisaging that this platform could individualize patient care and inform targeted resource allocation, particularly with regard to the frequency of patient medical appointments. In the context of the current COVID-19 pandemic, reducing face-to-face encounters without compromising clinical outcomes is of current interest to both patients and health professionals [12]. However, although digital innovation may offer solutions to problems, new systems must be accepted and regarded as advantageous to patients and worthwhile for providers to implement successfully in clinical practice [13].

### Objectives

We aim to develop a digital platform to facilitate the use of PROs in the context of chronic HIV infection. We believe that the meaningful engagement of health providers and patients, as equal stakeholders, will create a bidirectional incentive to use PRO software whereby “a common patient-centered frame of reference” [3] supports therapeutic communication and patient autonomy. Therefore, the digital communication package that we designed should reflect stakeholders’ collective and differing requirements. We opted to use an iterative development process of agile design and qualitative research methods [14-16], in contrast with earlier approaches to software design that, in targeting potential users, tended to focus on technological capacity and maximum specification.

We envisaged the software to support the following communication process: patients would input information at home or in the clinic before their appointments using their own devices. The software then transmits the information to the medical practice software through a secure portal. Using a custom interface, doctors could review concise data from an automated, preprocessed synthesis of patient self-reports during patient consultation. The longitudinal information displayed graphically provides clinicians with trends over time. We set the following objectives: (1) to design a web-based application compatible with mobile and desktop, (2) to assess comprehension of the platform, and perceived benefits, (3) to assess the usability of the designed product, and (4) to conduct a pilot study using PRO instruments integrated into the platform. Our interdisciplinary Australian and French teams included medical, psychology, information technology (IT), and digital communications experts, and the study was conducted in both countries with the teams holding regular teleconferences over the course of the study.

## Methods

### Study Design

The study was observational and iterative, with each activity informing the next. In stage 1, we created and tested a demonstration prototype or *wireframe* and assessed patient and clinician users’ comprehension of the rationale behind the software. We then refined the design further, loaded the software with two validated PRO instruments, the Depression, Anxiety and Stress Scale (DASS-21) [17] and the Patient Reported Outcomes Quality of Life-HIV (PROQOL-HIV) HRQL instrument specific for HIV [18]; in stage 2, we initiated a pilot study. The project was conducted between December 2015 and May 2017, and the Murdoch University Human Research Ethics Committee (2015/228) and the French Ethics Committee Ile de France IV–Institutional Review Board 00003835 (2016/44NI) approved the study. The research was conducted in 3 hospitals in France and 2 specialist HIV practices in Western Australia (WA). Members of the research team with fluency in both languages carried out translations when necessary.

### Participants

Participants were a sample of convenience and were eligible for the study if they were at least 18 years old and able to provide informed consent. All patients had been diagnosed with HIV infection and presented with a wide range of clinical and sociodemographic characteristics. Clinicians were trained either in infectious diseases or immunology, or were general practitioners with training in HIV medicine. Participants in stage 1 included patients with chronic HIV infection (Australian=4, French=36) and clinicians (Australian=4, French=12); those in stage 2 were patients (Australian=41, French=39) recruited from 3 hospitals in Paris and 2 community-based HIV specialist centers in WA. In addition, colleagues with various professional backgrounds, including clinicians, from within the 2 research organizations were asked to carry out the initial user testing of a basic prototype (stage 1).

### Data Analysis

An interview guide was developed collaboratively. One-on-one face-to-face interviews, up to 45 minutes in length, were conducted in the study hospitals in France and at specialist practices in Australia. The interviews were recorded and transcribed. The transcripts were imported to NVivo 12 (QSR International Pty Ltd.) and analyzed independently by a male master’s student, a female PhD student, and their PhD supervisors (SH, MD). An inductive thematic approach [19], informed by field data, was used to analyze the interview data. We coded text from the interviews to common themes and continued until the data were considered saturated [20]. Questionnaire data from the pilot study were exported and encrypted from the web application to the data capture program REDCap (Research Electronic Data Capture) [21] and subsequently downloaded into the software package R for quantitative analysis [22]. This included the derivation of descriptive summaries and the application of linear or logistic regression to explore associations with aspects of usability. Demographic information is presented as percentages or means, as appropriate.

### Stage 1: Design and Testing of the Initial Prototype

To approach the design of the software, we implemented a user-centered design [23] and agile development principles [14,15] that promote adaptive planning, evolutionary development, early delivery, and continuous improvement. This methodology provided increased transparency during the development phase through continuous feedback and flexibility when changes in direction were required. Our agile approach was informed by narratives, and as such, we educated the team on the lived patient experience of people affected by HIV by writing fictional personas informed by previous qualitative research [1].

**Figure 1:**
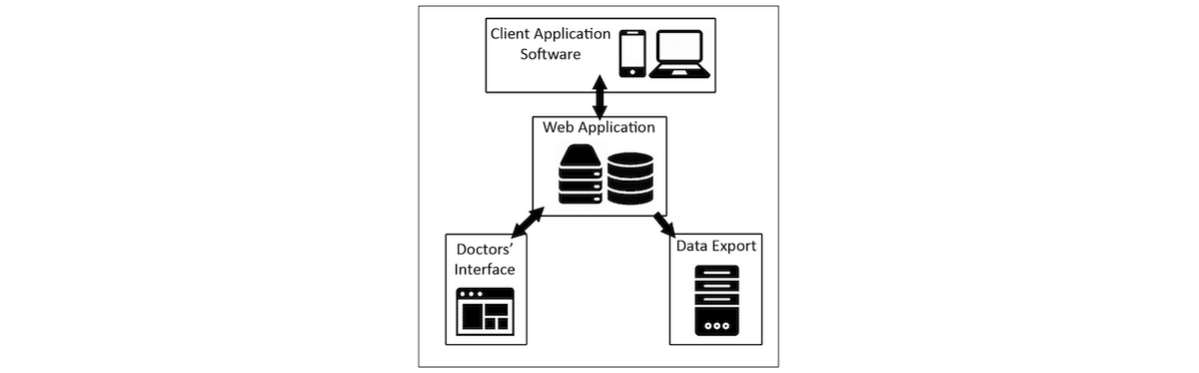
Diagram of communication between platforms.

The prototype consisted of basic functions, representing a minimum viable product. The architecture (Figure 1) comprises a web application that allowed qualitative instruments to be configured and patient accounts to be created. The instruments can then be deployed to client software applications that run on either mobile or desktop platforms and allow patients to provide responses to questionnaires. The client then submits the patient’s response data via a secure channel back to the web application, where it is stored in a database. The doctors’ interface was accessed through a web browser. This allows doctors and clinicians to access processed graphical reports of the scored patient responses to a PRO, identify longitudinal trends, and promote discussion with the patient. Finally, data export functionality allows the web application to export data in suitable formats for import into clinical patient management software. This architecture was implemented as a first pass to provide a minimum viable product for the initial testing.

We invited 20 colleagues, including clinicians, from both teams to use and comment on the software, which was configured with 10 testing items comprising questions and response categories. A member of the research team observed and recorded the users’ verbal responses as they navigated the application and administered a short questionnaire. The data were collated as *first round, in-house user testing* to improve the usability of the prototype.

### Acceptability of the Concept

Contemporaneously, we conducted 12 semidirected interviews of French patients to establish patients’ perceptions on using health care–related software and the difference eHealth might make to their care. Other questions were related to the current style of communication between patients and clinicians and their experiences with electronic devices and mobile health (mHealth) apps.

### Testing of the Prototype

The second round of user testing, comprising 44 semidirected interviews, took place in 3 Paris hospitals and 1 community-based clinic in WA. An interview guide was developed. In the first part of the interview, researchers described the purpose of the application and asked the patients (n=28) and doctors (n=16) about their computer usage habits and capabilities and their views on perceived benefits or harms of the software. They were then given an opportunity to view the application, and their reactions were noted [24]. Through this before-and-after approach, we were able to identify discrepancies between participants’ expectations of the software and subsequent user experience.

### Graphic Design

**Figure 2:**
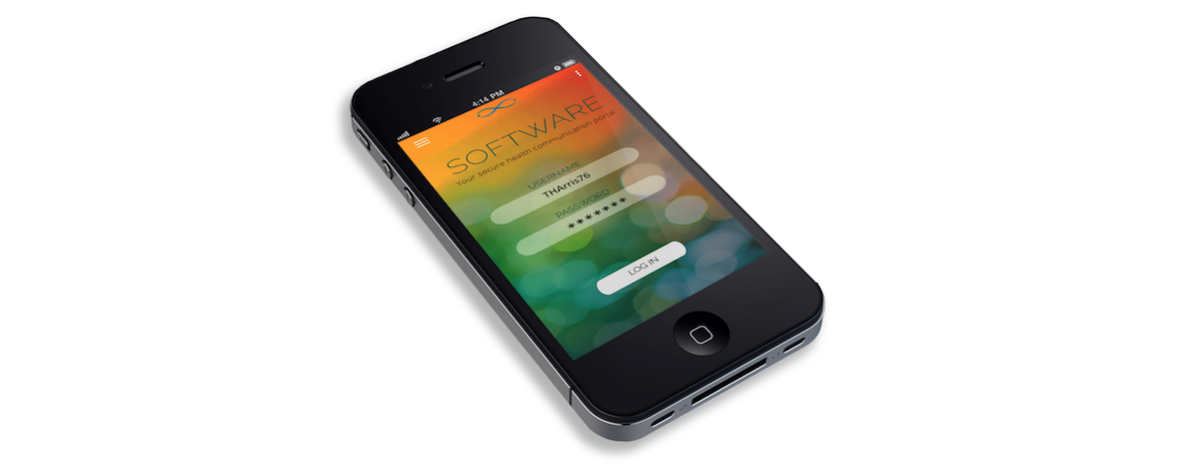
App design.

Informed by the fictional personae and predesign interviews, *mood boards* were created for colors, icons, and text (Multimedia Appendix 1). The initial design was for mobile devices such as smartphones, with the small screen presenting the greater design challenge regarding functionality. However, on a larger screen, there was no loss of function or esthetics (Figure 2). We used the composite data, empirical observations, previous research, and preliminary in-house user testing to create *user stories*, with short statements representing user requirements in French and English, which justified new features (Textbox 1). Each user story was coded to track the origin of the story from patient interview or empirical data (eg, “As a <stakeholder type> I want <some feature> so that <some benefit>”). Expressed in this way, it made the features easier to talk about and remember who they were for and to prioritize feature development. The stakeholders were patients, clinicians, researchers, and developers. The list of user stories informed a user story “backlog”—like a waiting room, ready to inform sprints that denoted a period of software development.

**User stories and sample quotes.**

**Sample Quotes**

- “As a patient, I want to be in control of my data, and know it is secure and private, so that I can feel comfortable providing it.”
- “As a patient, I want this software available on the types of device(s) I own, so that it is accessible to me.”
- “As a patient, I want clean, distraction free interaction with appropriate detail for fine control, so that my data are precise.”
- “As a patient, I want to use the software intuitively, without excessive instruction, so that it feels easy and effortless.”
- “As a patient, I want a personalized experience, so that it does not feel like filling out a form.”
- “As a doctor, I want clear and succinct results that inform a consultation, so that I do not get overwhelmed.”
- “As a doctor, I want unbiased results from the digital instruments, so that the patient reported outcomes are accurate.”

### Design of the Clinician Interface

In Australia, we interviewed 4 doctors, comprising 2 men and 2 women; 3 were from HIV specialist practices and 1 was hospital-based. We interviewed them before and after showing them the clinician interface (wireframe). The doctors were asked to envisage the utility of the software for measurement of HRQL and, informed by this feedback, the graphic artist refined the design of the interface (Figure 2).

### Stage 2: Pilot Studies in Australia and France

#### Australia

We recruited 41 Australians who were undergoing treatment for HIV infection from 2 high-case load community practices by the research nurse. After receiving minimal instruction and choosing a device (ie, desktop, laptop, tablet, or smartphone), they created an account and accessed the platform. Several participants preferred to use the software in their homes after phone contact with the researcher, but most accessed it in the clinic. The platform was configured using 4 questionnaires, including the 2 validated instruments: DASS-21 [17] and PROQOL-HIV [18,25]. The results of the 2 PROs are reported in this manuscript. Participants verbalized their experience while navigating the software to enable the researcher to note their observations, and they were instructed to record *true responses*. Participants also completed a usability questionnaire, and the researcher ranked the frequency of repetitive (similar) comments and provided a report to the developer.

#### France

The implementation of the pilot in France was similar to that in Australia. Participants included 39 outpatients from 2 centers, comprising 20 from the Paris area hospital, Kremlin Bicetre, and 19 in Saint Nazare, a regional hospital. Before the consultation, a research assistant introduced the study, and patients completed the questionnaires using a tablet or computer before consulting the doctor. Comments from this observation process and from participants were noted and synthesized.

## Results

### Stage 1: Design and Testing of the Initial Prototype

#### Sociodemographic Data

The sociodemographic background of the patient participants in France and Australia included in the study were different. The participants in Australia were predominantly White men, whereas 42% (10/24) of the participants in France were women, and in general, were more culturally and linguistically diverse, with 67% (16/24) from the African continent. All participants completed at least secondary level education.

#### Acceptability of the Concept: Patients’ Perspectives of Using Digital Health Technology

##### French Participants

Semidirected interviews were conducted with 24 patients in France, of which 92% (22/24) owned a computer, a third used a tablet, and half possessed smartphones. These interviews included 12 participants who had not seen the application and an additional 12 who reviewed the application after a demonstration using the wireframes to explain the features, by the researcher.

The use of mHealth and digital information sources among these respondents consisted mainly of fitness trackers, government services, and HIV information or news sites. Discussions included the concept of using mHealth devices to communicate HRQL issues with their doctors. Although 75% (18/24) of patients foresaw advantages to the application as described to them, the other 25% (6/24) could not see the point of using technology in place of face-to-face communication. Some respondents liked the idea of being able to contact their doctors directly, especially in an *emergency*, and 17% (4/24) did this through email. Others saw opportunities for the application to act as a reminder, thus focusing the consultation on the topics important to them. For example, one patient stated, “Sometimes when we meet, we do not remember what we wanted to say.” Another respondent thought it would strengthen engagement between doctor and patient:

In the health domain it will be a good tool...will bring patients closer to their doctors, it’s great...

However, for some participants, an mHealth evaluation of *quality of life* was not of particular value. One participant stated the following:

I do not see any utility, I am already followed, I have already been HIV positive for several years and I do the things (sic) well, it is well controlled. I have an appointment every six months. During the six months I do not see any usefulness of an application.

Some patients thought that the volume of data generated would overload their doctors and reduce the consultation time available for personal conversation. With regard to confidentiality, one patient described himself as “suspicious” of “these types of things.” An overriding concern for 45% (11/24) of French participants, particularly those with migrant backgrounds, was data security and confidentiality. One participant stated as follows:

You do not know to whom you can share all your life, you spread all your life like that; after you do not know who can recover this..., all that is not necessarily good.

The concern was that their information would be used in newspapers, and they would be identified and exposed to stigma and discrimination, or that information would be revealed if devices were lost. Our conclusion from this round of testing was that patients’ acceptance of a potential digital health care communication pathway in France was low.

##### Australian Participants

In Australia, 4 in-depth interviews were conducted. The comments of the participants before being shown the prototype application were similar to those of the French participants but reflected more optimism about eHealth services and less concern about confidentiality. In contrast to French participants, Australian participants used the prototype application and progressed through a series of questions drawn from the HRQL questionnaire. The screen design was minimal, showing one question per screen, and was accompanied by a *peaceful* soundtrack. The participants’ feedback after using the software was largely positive, and participants envisaged that doctors would have a better picture of their patients’ current health state if the information derived from the HRQL instrument was actively engaged with during the consultation; here, the caveat was how the general practitioner interpreted the patient information. There was a perception that digital communication in health was inevitable with one patient stating as follows:

I think it is good...The whole world works on technology, so the medical system may as well.

Another patient foresaw benefits to discourse within a prescribed timeframe: “It would be good for the software to be able to let me answer at my home at my own pace and [information] would be shared with my doctor.” However, one patient commented as follows:

OK, so you can get a prompt current reply with the technology, you don’t have to make an appointment to see a doctor, you can get it via technology.

The patient perceived this as a disadvantage that would potentially complicate his care, but the comment also suggested that the patient envisaged an electronic response from the doctor, rather than unidirectional communication, which was used to enhance face-to-face communication. Another patient considered his future clinical care:

I think for me, because I’m young and I don’t have a lot of effects from it [HIV] yet. I guess as I get older, I’m probably going to have more effects, so it’s able to keep a really good record of the effects on me, and the doctors always having that record, because it’s technology-based, it’s never going to go missing.

In contrast to French participants, concern about confidentiality among Australian participants was low. However, one participant would not complete the sensitive information on a portable device when the screen was visible to others. Vulnerability to hacking was also a concern, with a participant stating as follows:

It’s not so much you using the data or me giving the data to you...but if you’re ever hacked.

However, another participant said as follows:

No, I am not worried, I think confidentiality, like all software, is the same as on paper. You know if anyone wants to get into your documentation they need your passwords or something like that, so, it’s capable of doing that nowadays, if you’re doing banking and everything...

#### Design and Usability

The Australian group provided further detailed feedback about the design and usability of the software, commenting on the mouse versus touch screen and the navigation icons and their likes and dislikes. Consequently, the developer improved the product, removed the soundtrack, and added a privacy statement concerning the level of data encryption provided in the application.

#### Clinicians’ Perspectives: France and Australia

We deduced from our interviews with clinicians (12 in France and 4 in Australia) that three key design features were important. First, the PRO instrument chosen should be valid and disease specific. Second, it could be used to record and monitor outcomes of interventions and the impacts of the disease process. Finally, the application should be frictionless and integrated with the flow of communication between patient and clinician, as well as with other programs used simultaneously, which may require *screen switching*.

Younger clinicians had a more positive view of the potential use of mHealth applications than their older colleagues, and they valued digital clinical support software (eg, for prescribing medications). One doctor actively promoted mHealth apps to his patients for self-monitoring and motivational feedback. They also expressed interest in programs to increase retention and continuity of care, thus agreeing with the patient participants’ views on the application. One doctor said the following:

The benefit is certain...Because HIV patients are particular, [HIV] always has an impact on their lives, whether it is social or quality of life.

The 4 Australian doctors envisaged how it might be useful for chronic HIV management, as managed by nurses. They recognized the potential for integrating features to support the monitoring of treatment adherence and healthy lifestyle assessments (eg, drug and alcohol use).

#### Overwhelmed With Information

In general, doctors were concerned about information overload, with numerous complex concerns becoming difficult to address in the consultation time available. One doctor said, “...the data (for PROs) may be too complex for the average practitioner to interpret” and that, “an amateur assessment of exercise, for example, may not be better than no assessment of exercise.*”*

This clinician articulated that “doctors feel like they need to fix things,” implying that PROs might present them with information that could not be adequately actioned, leading to an unsatisfactory outcome for the patient. Another doctor said as follows:

It [the software] has to provide me with a reasonable summary of what is going on without taking 15 minutes to digest it. Otherwise, it just gets too much, does it provide value?...and people will not get through their work, and they will just ignore it.

French hospital-based doctors stressed that the time available within their consultations to review HRQL via a software application was extremely limited. In primary care, doctors tend to work within purpose-built IT systems for general practice. These programs often contain short questionnaires, such as an alcohol assessment; however, in hospitals, there is a pressing need for an integrated system of IT programs. One doctor stated as follows:

...there are a lot of applications that are not integrated...and you have to open up 5 different screens to access 5 different aspects of their (patients) care.

After seeing a representation of the doctors’ interface, the 3 primary care clinicians noted the utility of having patient-reported HRQL synthesized and particular items flagged for attention, thus saving time and facilitating what one doctor described as “a better structure to the consultation.” One doctor noted that the software could benefit patients with memory problems.

#### Patient Safety

One of the hospital-based clinicians was less convinced of the value of the software for individual patient care because patients’ queries are triaged by nurses, whereas in the primary care setting, communication is largely within the consultation and triaged by the doctor. Consequently, one doctor said, “it is very important for the patients not to consider that by pressing ‘send’ they are directly communicating urgently to the doctor.” Clinicians placed emphasis on the need to manage patient expectations and the necessity for clinician training to use and interpret PRO data, so that it can be actioned appropriately.

#### Confidentiality

In France, doctors were more likely to address the issue of data security rather than patient confidentiality. They considered *absolute security* as a critical element of the application. Doctors mentioned several potential risks related to the use of such an application, namely data storage on mobile devices, data submission via the internet, and data security in hospital servers.

#### Clinicians’ Interface

**Figure 3:**
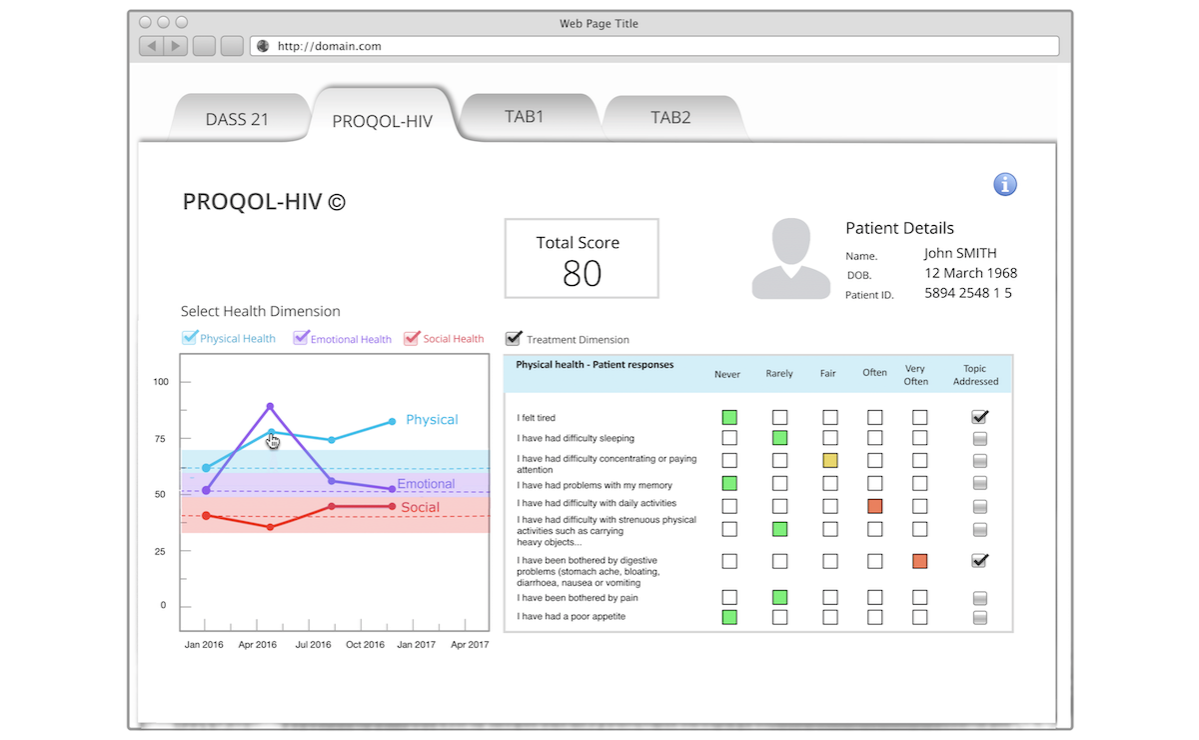
Clinicians’ interface.

The clinicians’ interface (Figure 3) addresses concerns about the volume of granular information generated by PRO instruments. We used the HRQL questionnaire to illustrate the function of the display for PRO data. Briefly, the items displayed on the screen are color-coded to each response (rarely, sometimes, always, etc), with the color red indicating patient concern to emphasize the report as patient-driven. Accordingly, a sidebar checkbox allows the doctor to indicate that a discussion has taken place. Sections pertaining to physical, emotional, and social health dimensions are displayed as graphs to enable the visualization of changes in health dimension scores over time.

### Stage 2: Results of the Pilot Studies in Australia and France

Table 1 shows the characteristics of patient participants in the pilot study. The average age of Australian patients was 57 years and 90% (37/41) were men, and HIV transmission was largely sexual reflecting the demographic of the Australian HIV epidemic [26]. The results of the usability testing (Table 2) showed that 98% (40/41) of the participants found the application easy to use, and 61% (25/41) comprehended it quickly. We also observed that older age was associated with reduced scores for understanding the visual interface (*P*=.004) and finding the *buttons* organized (*P*=.02). Furthermore, 24% (10/41) of respondents were concerned with confidentiality, and this result was more prevalent in participants with higher anxiety and stress scores, as measured by the DASS inventory (*P*=.007 and *P*=.01, respectively).

The French pilot patient participants’ median age was 53 years; they were mostly men and more often single. Usability testing was completed by 19 participants. The results were similar to that of Australian participants regarding ease of use and quick comprehension; however, the French participants were more concerned about confidentiality (9/19, 47%) than the Australian participants. However, for the 15 patients who completed the usability questionnaire and the DASS, the correlation between confidentiality and anxiety and stress scores measured by the DASS inventory was not significant (*r*=0.37; *P*=.17). The association between older Australians and concern about the organization and function of the application was not tested in the French participants because most French participants had assigned a maximum score to those items, creating a *ceiling effect*.

The commentary received from the French patients was largely positive for all aspects of the application design. Overall, patients reported being glad to identify topics that they would not usually or spontaneously discuss with their clinician, and this opportunity could incentivize them to use the application over a longer period. A concern was raised that standardization via the use of questionnaires may threaten individualized care and not take into consideration individual circumstances. For doctors, an annoyance was the necessity to open another software application on their screen, in addition to the patient record.

**Table 1:** Characteristics of patients in the pilot study (N=80).

Characteristics				Australian patients					French patients		
				Total, n (%)		Value		Total, n (%)			Value
**Demographics, median (range)**											
	Age (years)			41 (51)		58 (50-64)		39 (49)			52 (44-60)
	Duration HIV (months)			34 (43)		228 (153-312)		36 (45)			126 (69-240)
**Sex, n (%)**				41 (51)				36 (45)			
	Male					37 (90)					29 (81)
	Female					4 (10)					7 (19)
**Ethnicity, n (%)**				38 (48)				36 (45)			
	White					33 (87)					27 (75)
	Asian					4 (10)					1 (3)
	Middle Eastern					1 (3)					0 (0)
	African					0 (0)					7 (19)
	Latinx					0 (0)					1 (3)
**Mode of transmission, n (%)**				33(41)				36 (45)			
	MSM^a^					27 (82)					22 (61)
	Heterosexual					6 (18)					14 (39)
**Employment, n (%)**				41 (51)				19 (34)			
	Employed					23 (56)					12 (63)
	Not employed					18 (44)					7 (37)
**Clinical variables**											
	CD4 T cells, median (range)			31 (39)		703 (458-946)		36 (45)			610 (480-872)
	**HIV viral load, n (%)**										
		>20 copies/ml	31 (39)		N/A^b^		33 (41)			33 (92)	
		<20 copies/ml	31 (39)		31 (100)		3 (4)			3 (8)	
	HBV-HCV coinfection, n (%)			31 (39)		0 (0)		35 (44)			10 (29)

^a^MSM: men who have sex with men.

^b^N/A: Not applicable.

**Table 2:** Pilot study usability testing^a^ (N=60).

Response^b^	Australian (n=41), n (%)	French (n=19), n (%)
Easy to use	40 (98)	18 (95)
I would need support to use it	0 (0)	3 (16)
I would use it again	41 (100)	16 (84)
Had a clear, clean design	40 (98)	18 (95)
Required minimum screen changes	41 (100)	N/A^c^
I found it easy and quick to comprehend	25 (61)	18 (95)
The instructions were clear and unambiguous	37 (90)	17 (90)
The buttons organized and easy to find	35 (85)	19 (100)
I understood the function	28 (68)	19 (100)
I found it easy to navigate	40 (98)	19 (100)
The size, style, and font were appropriate	39 (95)	N/A
It was a pleasant experience	38 (93)	17 (90)
I found it intuitive to use	31 (76)	15 (79)
I was concerned about confidentiality	10 (24)	9 (47)

^a^Pilot test was carried out in a primary health care setting (Australia) and a hospital-based setting (France).

^b^Responses to the questionnaire item “The application was judged to be.”

^c^N/A: Not applicable (two items were not asked of French participants).

## Discussion

### Principal Findings

We designed and tested a software application enabling the transfer of PRO from a user’s device to a clinician’s electronic patient management system through a secure portal. A key capability is the display of synthesized data with the potential to focus patient and doctor discourse on patient-driven information; the data are measurable and can be mapped against the implementation of interventions (ie, action points). The design permits the prioritization of actions between visits, further supporting patient preference, and the use of validated instruments could accommodate concerns about the consistency and reliability of repeated PRO results. As the software is configurable, validated PRO instruments can be selected based on clinical requirements (eg, a questionnaire for monitoring side effects or adherence to a new medication). The use of ePRO measures is becoming commonplace and acceptable with some caveats [27]. Our methods enabled us to integrate users’ opinions on the application design and increase the validity of the product [28] and its visual appeal. The design is a combination of function and form, as it is esthetically pleasing. However, by taking a minimal approach to the software design, we maximized the potential of the product to meet the needs of the end user and prevent “overspecification.” Although we found that older age was associated with reduced scores for some aspects of usability, none of the patients in the pilot study indicated that they would need support to use the application again.

Data from the predesign phase indicated interest in enhanced patient-doctor communication, supported by mHealth strategies, although a few patients considered it unnecessary. Hitherto, most participants had only thought of mHealth in terms of health monitoring and fitness applications. There was initial confusion regarding what form the flow of information would take and what feedback, if any, they would receive from their doctors. The French patients considered feedback important and expected results or a *score*. The Australian patients were more concerned about how the doctor would use this information and whether it would improve their care in the long term. In general, participants envisaged an *efficiency gain* for themselves, and the doctors, if the volume of data transmitted was not overwhelming. The idea of entering data at home in preparation for their medical consult fits with their practice of a preappointment blood test, whereby the results would be ready for discussion on arrival. In both countries, electronic transmission of laboratory results to medical practice software and linkage with the patient’s file is common. Confidentiality and data security were major concerns among French patients, particularly migrants, and doctors. Such concerns were less striking among Australian interviewees who had confidence in data protection processes. However, the results of the Australian pilot study revealed that participants with high stress and anxiety scores as measured by the DASS tended to worry about confidentiality when entering their own health information in actuality, as opposed to a hypothetical situation, and this was true of the French participants as well. Evidence also showed that participants perceived greater privacy in using the application on a computer in their own home, in contrast to using the application on a mobile device in public. Notwithstanding, people with stigmatized illnesses are significantly more likely to use the internet to communicate with clinicians [29].

### Strengths and Limitations of the Study

We conducted our study in a primary care context in Australia and a hospital-based setting in France. Although this is a strength in the context of product development, it has implications for the design of randomized controlled trials and the subsequent implementation of PRO measures in clinical care [30,31]. We tested our software in the context of HIV care, but we believe our findings are equally useful for other chronic illnesses. Both countries offer free universal health care; however, in Australia, primary care is fee for service and government rebates rarely cover the cost of a consultation, which varies according to length. Public hospital care is free, but clinics are busy and there is an incentive for shorter consultations. The use of eHealth software to support the integration of PRO could reduce the number of doctor visits, as reported by Kjaer et al [10] without compromising patient care. In addition, for web-based platforms used in health settings and within most practice, software has the ability to import data from other programs via interoperable formats such as XML. We achieved this using REDCap as a proxy for practice software. This served as a good test case for import and export functionalities, and the software was designed so that developing and plugging export adaptors for specific and proprietary clinical software packages is possible.

Our software was tested in a research environment, and although the esthetics were carefully considered to make it pleasurable to use, we do not know if patients will experience software *fatigue* over time, as is evidenced by the large number of mobile apps that have been designed [32]. However, we believe that a design that facilitates an experience that feels personal, in contrast with a well-constructed but impersonal form, will influence a patient’s perception of the quality of care they receive in the absence of direct contact; this will be advantageous for people living in rural and remote settings. Our participants were largely computer literate, which may affect the generalizability of the product suitability for other demographic groups. In addition, some might express concern that variation in the mode of delivery might affect responses; however, research has found that when sampling protocols are followed, data equivalence can be achieved, although this research came with caveats [33]. The doctors also showed interest in using the software; however, further work will be necessary, as well as education and training on the utility of PRO data per se for patient care, as has been flagged by others [27,34]. A systematic review of mHealth adoption by Gagnon et al [35] found that utility and ease of use were two of the most important factors influencing uptake by health care professionals, and more recent reviews [30] reported organizational factors that must be considered. However, these factors vary according to the context.

### Conclusions

We recognize that to be an effective tool and to provide clear value, the therapeutic benefit of using this application needs to be evident to doctors and patients [16]. This could be considered both a strength and a weakness of our design; however, the concept of the therapeutic relationship between clinician and patient underpins our design. For example, interaction with the doctor during the consultation could motivate the patient to input PRO data at home in preparation for the next visit, and equally, the clinical *return* achieved will motivate the doctor to use the software repeatedly. We also see the potential for the integration of a range of PRO instruments in a single platform, not only confined to the management of HIV infection, including, for example, side-effect questionnaires. These could help quantify the qualitative impacts of pharmaceutical interventions in tandem with medication adherence between visits. To establish the utility of this application, we envisage that a randomized controlled trial design, informed by an implementation strategy and coupled with a qualitative analysis evaluating the software over an extended period, will be necessary.

## Appendix

Multimedia Appendix 1 Moodboard.

## Abbreviations

DASS: Depression, Anxiety and Stress Scale
HRQL: health-related quality of life
IT: information technology
mHealth: mobile health
PRO: patient-reported outcome
PROQOL-HIV: Patient Reported Outcomes Quality of Life-HIV
REDCap: Research Electronic Data Capture
WA: Western Australia
