# Supplementary figures and images for: Supporting Patient-Clinician Interaction in Chronic HIV Care: Design and Development of a Patient-Reported Outcomes Software Application
Source: J Med Internet Res. 2021 Jul 30;23(7):e27861. doi: 10.2196/27861 (PMC8367117; doi:10.2196/27861)

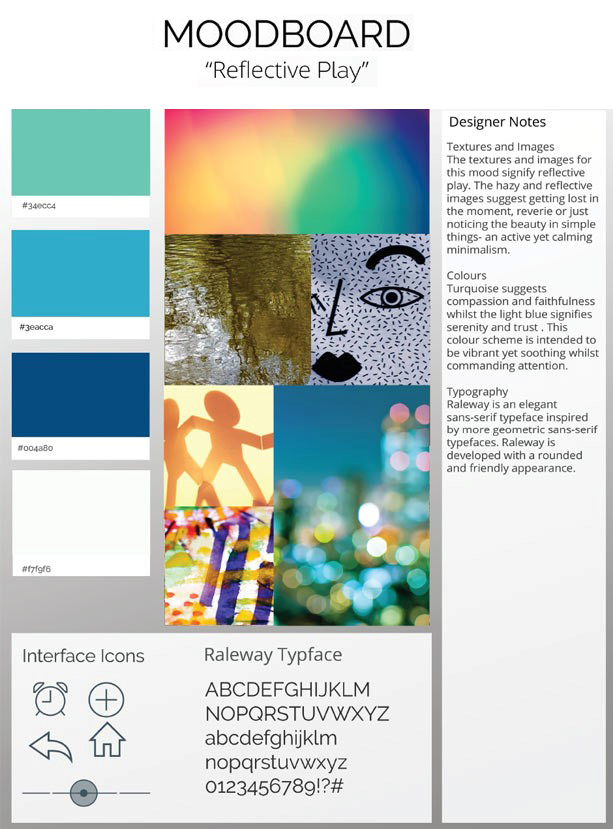


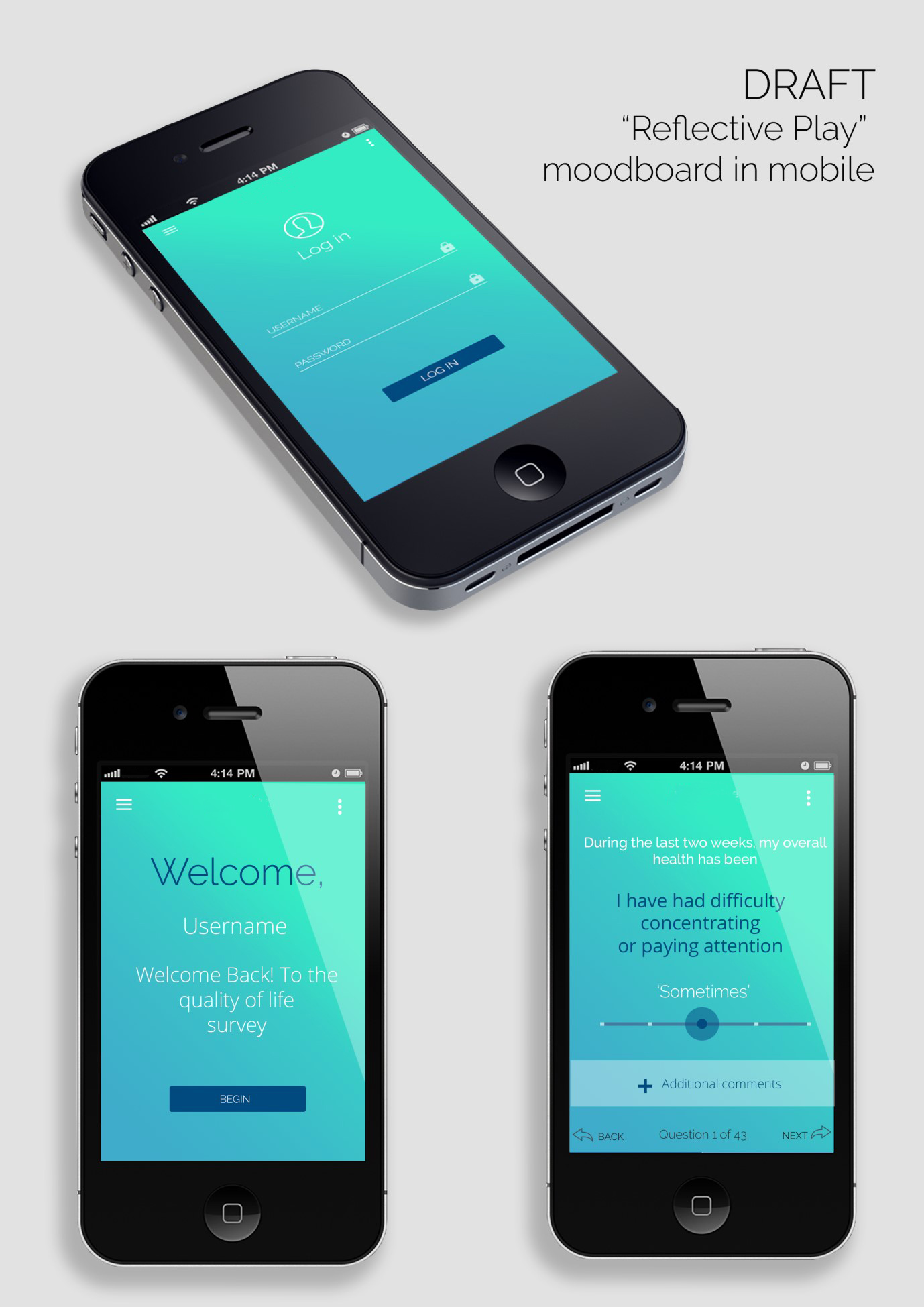

Supplement: Multimedia Appendix 1 [file jmir_v23i7e27861_app1.docx]
